# 4-Methyl­sulfanyl-6-(4-pyrid­yl)-1,3,5-triazin-2-amine

**DOI:** 10.1107/S1600536811013171

**Published:** 2011-04-16

**Authors:** Ya-Pan Wu, Long Tang, Feng Fu, Qi-Rui Liu

**Affiliations:** a Department of Chemistry and Chemical Engineering, Shaanxi Key Laboratory of Chemical Reaction Engineering, Yan’an University, Yan’an, Shaanxi, 716000, People’s Republic of China

## Abstract

In the title compound, C_9_H_9_N_5_S, the pyridyl and triazine rings make a dihedral angle of 4.8 (2)°. In the crystal, adjacent mol­ecules are bridged by an N—H⋯N hydrogen bond, forming a helical chain running along the *b* axis.

## Related literature

For the use of *N*-heterocycles in the synthesis of solid-state architectures, see: Janczak *et al.* (2003 ▶). For silmilar triazine derivatives, see: Ma & Che (2003 ▶).
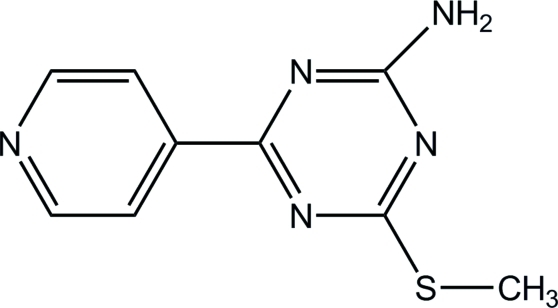


## Experimental

### 

#### Crystal data

C_9_H_9_N_5_S
*M* *_r_* = 219.27
Orthorhombic, 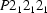

*a* = 3.9002 (11) Å
*b* = 10.111 (3) Å
*c* = 25.143 (7) Å
*V* = 991.4 (5) Å^3^
*Z* = 4
Mo *K*α radiation
μ = 0.30 mm^−1^
*T* = 298 K
0.29 × 0.08 × 0.06 mm

#### Data collection

Bruker SMART diffractometer
Absorption correction: multi-scan (*SADABS*; Sheldrick, 1996 ▶) *T*_min_ = 0.919, *T*_max_ = 0.982
5053 measured reflections
1083 independent reflections
877 reflections with *I* > 2σ(*I*)
*R*_int_ = 0.055

#### Refinement

*R*[*F* ^2^ > 2σ(*F* ^2^)] = 0.046
*wR*(*F* ^2^) = 0.096
*S* = 1.06
1083 reflections
136 parameters
H-atom parameters constrained
Δρ_max_ = 0.24 e Å^−3^
Δρ_min_ = −0.24 e Å^−3^

### 

Data collection: *SMART* (Bruker, 1997 ▶); cell refinement: *SAINT* (Bruker, 1997 ▶); data reduction: *SAINT*; program(s) used to solve structure: *SHELXS97* (Sheldrick, 2008 ▶); program(s) used to refine structure: *SHELXL97* (Sheldrick, 2008 ▶); molecular graphics: *SHELXTL* (Sheldrick, 2008 ▶); software used to prepare material for publication: *SHELXTL*.

## Supplementary Material

Crystal structure: contains datablocks I, global. DOI: 10.1107/S1600536811013171/ng5131sup1.cif

Structure factors: contains datablocks I. DOI: 10.1107/S1600536811013171/ng5131Isup2.hkl

Additional supplementary materials:  crystallographic information; 3D view; checkCIF report

## Figures and Tables

**Table 1:** Hydrogen-bond geometry (Å, °)

*D*—H⋯*A*	*D*—H	H⋯*A*	*D*⋯*A*	*D*—H⋯*A*
N4—H4*A*⋯N5^i^	0.86	2.10	2.956 (4)	172

Symmetry code: (i) 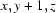.

**Fig. 1.**
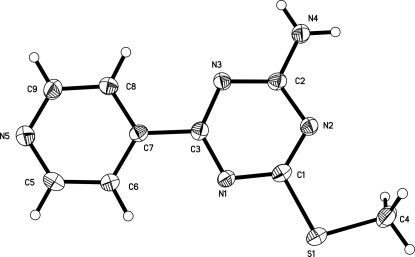
The molecular structure and labeling of (I). Displacement ellipsoids are drawn at the 30% probability level.

**Fig. 2.**
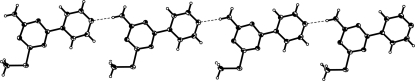
The one-dimensional linear structre (I), viewed along the b axis via N—H···N hydrogen bonding interaction. Dashed lines denote hydrogen bonds.

## supplementary crystallographic information

### Comment

The title compound (I), (Fig. 1), crystallizes in space group P2(1)2(1)2(1) with one crystallographically independent molecule per asymmetric unit.Strong intermolecular N—H···N hydrogen bonds link the subunit into the one-dimensional linear structure (Fig. 2).

### Experimental

The title compound was crystallized by hydrothermal method. A mixture of Cu(Ac)_2_.H_2_O (0.20 mmol), 2-amino-4-methylthio-6-(4-pyridyl)- 1,3,5-triazine (ampt, 0.20 mmol), 3-(4-carboxyphenyl)propionic acid (H_2_cppa 0.20 mmol) 0.2*M* NaOH (0.1 mL) and water (10 ml) was stirred for 20 min. The mixture was then transferred to a 23 ml Teflon-lined autoclave and kept at 413 K for 72 h under autogenous pressure. Then the mixture was cooled to room temperature slowly. The targeted ternary Cu(II) coordination polymer was not synthesized. Accidentally,the compound ampt was returned unchangedly and crystallized in the hydrothermal reaction.Finally, Colorless single crystals of the title compound suitable for X-ray analysis were obtained from the reaction mixture.

### Refinement

The C-bound H atoms were geometrically placed (C—H = 0.93 Å) and refined as riding with *U*_iso_(H) =1.2*U*_eq_(C). The NH H atoms were found from a difference Fourier map and restrained to 0.86 Å, and refined with *U*_iso_(H) =1.2*U*_eq_(N). Friedel equivalents have been merged.

### Crystal data

**Table:**

C_9_H_9_N_5_S	*F*(000) = 456
*M**_r_* = 219.27	*D*_x_ = 1.469 Mg m^−^^3^
Orthorhombic, *P*2_1_2_1_2_1_	Mo *K*α radiation, λ = 0.71073 Å
Hall symbol: P 2ac 2ab	θ = 1.6–25.0°
*a* = 3.9002 (11) Å	µ = 0.30 mm^−^^1^
*b* = 10.111 (3) Å	*T* = 298 K
*c* = 25.143 (7) Å	Prism, colorless
*V* = 991.4 (5) Å^3^	0.29 × 0.08 × 0.06 mm
*Z* = 4	

### Data collection

**Table:**

Bruker SMART diffractometer	1083 independent reflections
Radiation source: fine-focus sealed tube	877 reflections with *I* > 2σ(*I*)
graphite	*R*_int_ = 0.055
φ and ω scans	θ_max_ = 25.0°, θ_min_ = 1.6°
Absorption correction: multi-scan (*SADABS*; Sheldrick, 1996)	*h* = −4→4
*T*_min_ = 0.919, *T*_max_ = 0.982	*k* = −10→12
5053 measured reflections	*l* = −29→29

### Refinement

**Table:**

Refinement on *F*^2^	Primary atom site location: structure-invariant direct methods
Least-squares matrix: full	Secondary atom site location: difference Fourier map
*R*[*F*^2^ > 2σ(*F*^2^)] = 0.046	Hydrogen site location: inferred from neighbouring sites
*wR*(*F*^2^) = 0.096	H-atom parameters constrained
*S* = 1.06	*w* = 1/[σ^2^(*F*_o_^2^) + (0.0478*P*)^2^ + 0.2208*P*] where *P* = (*F*_o_^2^ + 2*F*_c_^2^)/3
1083 reflections	(Δ/σ)_max_ = 0.001
136 parameters	Δρ_max_ = 0.24 e Å^−^^3^
0 restraints	Δρ_min_ = −0.24 e Å^−^^3^

### Special details

**Table:**

Geometry. All e.s.d.'s (except the e.s.d. in the dihedral angle between two l.s. planes) are estimated using the full covariance matrix. The cell e.s.d.'s are taken into account individually in the estimation of e.s.d.'s in distances, angles and torsion angles; correlations between e.s.d.'s in cell parameters are only used when they are defined by crystal symmetry. An approximate (isotropic) treatment of cell e.s.d.'s is used for estimating e.s.d.'s involving l.s. planes.
Refinement. Refinement of *F*^2^ against ALL reflections. The weighted *R*-factor *wR* and goodness of fit *S* are based on *F*^2^, conventional *R*-factors *R* are based on *F*, with *F* set to zero for negative *F*^2^. The threshold expression of *F*^2^ > σ(*F*^2^) is used only for calculating *R*-factors(gt) *etc*. and is not relevant to the choice of reflections for refinement. *R*-factors based on *F*^2^ are statistically about twice as large as those based on *F*, and *R*- factors based on ALL data will be even larger.

### Fractional atomic coordinates and isotropic or equivalent isotropic displacement parameters (Å^2^)

**Table:**

	*x*	*y*	*z*	*U*_iso_*/*U*_eq_	
N1	1.1007 (9)	0.9098 (3)	0.16248 (11)	0.0339 (8)	
N2	1.0449 (9)	1.1369 (3)	0.13727 (11)	0.0343 (9)	
N3	0.8318 (10)	0.9659 (3)	0.08066 (11)	0.0351 (8)	
N4	0.7949 (11)	1.1828 (3)	0.05641 (13)	0.0478 (11)	
H4A	0.8298	1.2657	0.0620	0.057*	
H4B	0.6971	1.1574	0.0275	0.057*	
N5	0.8411 (12)	0.4703 (3)	0.07660 (13)	0.0495 (10)	
S1	1.3341 (3)	1.07833 (10)	0.23066 (4)	0.0426 (3)	
C1	1.1382 (10)	1.0410 (4)	0.17022 (13)	0.0329 (9)	
C2	0.8925 (11)	1.0938 (4)	0.09250 (14)	0.0339 (10)	
C3	0.9465 (10)	0.8803 (3)	0.11698 (14)	0.0311 (10)	
C4	1.3782 (12)	1.2539 (4)	0.22817 (16)	0.0508 (12)	
H4C	1.4850	1.2846	0.2603	0.076*	
H4D	1.1559	1.2938	0.2247	0.076*	
H4E	1.5175	1.2779	0.1982	0.076*	
C5	0.9850 (14)	0.5082 (4)	0.12212 (17)	0.0489 (13)	
H5	1.0671	0.4430	0.1449	0.059*	
C6	1.0198 (13)	0.6385 (4)	0.13752 (15)	0.0432 (12)	
H6	1.1208	0.6594	0.1699	0.052*	
C7	0.9028 (10)	0.7375 (3)	0.10425 (13)	0.0309 (9)	
C8	0.7467 (11)	0.6986 (4)	0.05768 (15)	0.0377 (11)	
H8	0.6563	0.7616	0.0346	0.045*	
C9	0.7248 (12)	0.5655 (4)	0.04524 (16)	0.0460 (12)	
H9	0.6230	0.5417	0.0132	0.055*	

### Atomic displacement parameters (Å^2^)

**Table:**

	*U*^11^	*U*^22^	*U*^33^	*U*^12^	*U*^13^	*U*^23^
N1	0.040 (2)	0.0343 (19)	0.0279 (17)	−0.0042 (17)	−0.0046 (16)	−0.0005 (13)
N2	0.041 (2)	0.0336 (17)	0.0289 (18)	−0.0003 (17)	0.0012 (17)	−0.0010 (14)
N3	0.049 (2)	0.0304 (17)	0.0264 (17)	0.0020 (18)	−0.0011 (18)	0.0006 (13)
N4	0.076 (3)	0.0345 (17)	0.0325 (19)	−0.003 (2)	−0.011 (2)	0.0013 (15)
N5	0.066 (3)	0.0368 (18)	0.046 (2)	−0.004 (2)	−0.010 (2)	0.0011 (16)
S1	0.0492 (6)	0.0466 (6)	0.0318 (5)	−0.0024 (6)	−0.0092 (5)	−0.0037 (4)
C1	0.027 (2)	0.043 (2)	0.028 (2)	−0.002 (2)	0.001 (2)	−0.0035 (17)
C2	0.040 (3)	0.038 (2)	0.0235 (19)	−0.001 (2)	0.0020 (19)	0.0013 (18)
C3	0.035 (2)	0.034 (2)	0.025 (2)	−0.0040 (19)	0.0058 (19)	−0.0016 (16)
C4	0.056 (3)	0.053 (3)	0.044 (2)	−0.002 (3)	−0.010 (3)	−0.015 (2)
C5	0.059 (3)	0.042 (3)	0.046 (3)	0.002 (2)	−0.009 (3)	0.0127 (19)
C6	0.055 (3)	0.038 (2)	0.036 (2)	−0.005 (2)	−0.013 (2)	0.0021 (19)
C7	0.031 (2)	0.037 (2)	0.0238 (19)	−0.002 (2)	0.0028 (19)	0.0000 (17)
C8	0.044 (3)	0.037 (2)	0.032 (2)	0.000 (2)	−0.002 (2)	0.0039 (16)
C9	0.060 (3)	0.041 (2)	0.037 (2)	−0.009 (3)	−0.005 (2)	−0.0029 (19)

### Geometric parameters (Å, °)

**Table:**

N1—C3	1.327 (5)	C3—C7	1.488 (5)
N1—C1	1.349 (4)	C4—H4C	0.9600
N2—C1	1.326 (4)	C4—H4D	0.9600
N2—C2	1.345 (4)	C4—H4E	0.9600
N3—C3	1.336 (5)	C5—C6	1.379 (5)
N3—C2	1.348 (4)	C5—H5	0.9300
N4—C2	1.333 (5)	C6—C7	1.383 (5)
N4—H4A	0.8600	C6—H6	0.9300
N4—H4B	0.8600	C7—C8	1.377 (5)
N5—C9	1.325 (5)	C8—C9	1.384 (5)
N5—C5	1.331 (5)	C8—H8	0.9300
S1—C1	1.742 (4)	C9—H9	0.9300
S1—C4	1.785 (4)		
			
C3—N1—C1	113.3 (3)	H4C—C4—H4D	109.5
C1—N2—C2	114.1 (3)	S1—C4—H4E	109.5
C3—N3—C2	114.4 (3)	H4C—C4—H4E	109.5
C2—N4—H4A	120.0	H4D—C4—H4E	109.5
C2—N4—H4B	120.0	N5—C5—C6	123.9 (4)
H4A—N4—H4B	120.0	N5—C5—H5	118.0
C9—N5—C5	116.5 (3)	C6—C5—H5	118.0
C1—S1—C4	103.12 (19)	C5—C6—C7	119.3 (4)
N2—C1—N1	126.8 (3)	C5—C6—H6	120.3
N2—C1—S1	120.5 (3)	C7—C6—H6	120.3
N1—C1—S1	112.7 (3)	C8—C7—C6	117.0 (4)
N4—C2—N2	118.5 (3)	C8—C7—C3	120.7 (3)
N4—C2—N3	116.6 (3)	C6—C7—C3	122.3 (3)
N2—C2—N3	124.9 (3)	C7—C8—C9	119.8 (4)
N1—C3—N3	126.5 (3)	C7—C8—H8	120.1
N1—C3—C7	117.1 (3)	C9—C8—H8	120.1
N3—C3—C7	116.3 (3)	N5—C9—C8	123.4 (4)
S1—C4—H4C	109.5	N5—C9—H9	118.3
S1—C4—H4D	109.5	C8—C9—H9	118.3
			
C2—N2—C1—N1	1.3 (6)	C2—N3—C3—C7	−176.7 (4)
C2—N2—C1—S1	−179.1 (3)	C9—N5—C5—C6	−0.5 (8)
C3—N1—C1—N2	−1.5 (6)	N5—C5—C6—C7	−0.5 (8)
C3—N1—C1—S1	178.9 (3)	C5—C6—C7—C8	1.9 (7)
C4—S1—C1—N2	−3.7 (4)	C5—C6—C7—C3	−177.0 (4)
C4—S1—C1—N1	176.0 (3)	N1—C3—C7—C8	−179.7 (4)
C1—N2—C2—N4	−178.8 (4)	N3—C3—C7—C8	−1.0 (6)
C1—N2—C2—N3	0.6 (6)	N1—C3—C7—C6	−0.8 (6)
C3—N3—C2—N4	177.4 (4)	N3—C3—C7—C6	177.9 (4)
C3—N3—C2—N2	−2.0 (6)	C6—C7—C8—C9	−2.4 (6)
C1—N1—C3—N3	−0.3 (6)	C3—C7—C8—C9	176.6 (4)
C1—N1—C3—C7	178.3 (3)	C5—N5—C9—C8	0.0 (7)
C2—N3—C3—N1	1.9 (6)	C7—C8—C9—N5	1.5 (7)

### Hydrogen-bond geometry (Å, °)

**Table:**

*D*—H···*A*	*D*—H	H···*A*	*D*···*A*	*D*—H···*A*
N4—H4A···N5^i^	0.86	2.10	2.956 (4)	172

Symmetry codes: (i) *x*, *y*+1, *z*.
